# Practical whole-tooth restoration utilizing autologous bioengineered tooth germ transplantation in a postnatal canine model

**DOI:** 10.1038/srep44522

**Published:** 2017-03-16

**Authors:** Mitsuaki Ono, Masamitsu Oshima, Miho Ogawa, Wataru Sonoyama, Emilio Satoshi Hara, Yasutaka Oida, Shigehiko Shinkawa, Ryu Nakajima, Atsushi Mine, Satoru Hayano, Satoshi Fukumoto, Shohei Kasugai, Akira Yamaguchi, Takashi Tsuji, Takuo Kuboki

**Affiliations:** 1 Department of Oral Rehabilitation and Regenerative Medicine, Graduate School of Medicine, Dentistry and Pharmaceutical Sciences, Okayama University, Okayama, 700-8525, Japan; 2 Department of Molecular Biology and Biochemistry, Graduate School of Medicine, Dentistry and Pharmaceutical Sciences, Okayama University, Okayama, 700-8525, Japan; 3 Research Institute for Science and Technology, Tokyo University of Science, Noda, Chiba, 278-8510, Japan; 4 RIKEN Center for Developmental Biology, Kobe, Hyogo, 650-0047, Japan; 5 Organ Technologies Inc., Tokyo, 105-0001, Japan; 6 Department of Fixed Prosthodontics, Osaka University Graduate School of Dentistry, Yamadaoka, Suita, Osaka 565-0871, Japan; 7 Department of Orthodontics, Graduate School of Medicine, Dentistry and Pharmaceutical Sciences, Okayama University, Okayama, 700-8525, Japan; 8 Division of Pediatric Dentistry, Tohoku University Graduate School of Dentistry, Sendai, Miyagi, 980-8575, Japan; 9 Section of Oral Implantology and Regenerative Dental Medicine, Graduate School of Tokyo Medical and Dental University, Bunkyo-ku, Tokyo, 113-8549, Japan; 10 Section of Oral Pathology, Department of Oral Restitution, Graduate School of Tokyo Medical and Dental University, Chiyoda-ku, Tokyo, 113-8549, Japan; 11 Oral Health Science Center, Tokyo Dental College, Chiyoda-ku, Tokyo, 101-0061, Japan

## Abstract

Whole-organ regeneration has great potential for the replacement of dysfunctional organs through the reconstruction of a fully functional bioengineered organ using three-dimensional cell manipulation *in vitro*. Recently, many basic studies of whole-tooth replacement using three-dimensional cell manipulation have been conducted in a mouse model. Further evidence of the practical application to human medicine is required to demonstrate tooth restoration by reconstructing bioengineered tooth germ using a postnatal large-animal model. Herein, we demonstrate functional tooth restoration through the autologous transplantation of bioengineered tooth germ in a postnatal canine model. The bioengineered tooth, which was reconstructed using permanent tooth germ cells, erupted into the jawbone after autologous transplantation and achieved physiological function equivalent to that of a natural tooth. This study represents a substantial advancement in whole-organ replacement therapy through the transplantation of bioengineered organ germ as a practical model for future clinical regenerative medicine.

Oral functions, including mastication, swallowing and pronunciation, are indispensable for adequate general health, social activity and quality of life^1^. These functions are carried out by the teeth, masticatory muscles and temporomandibular joint under the control of the central nervous system^2^,^3^. The tooth is an ectodermal organ whose development is regulated by reciprocal epithelial-mesenchymal interactions, and the tooth comprises both distinctive hard tissue (*e.g.*, enamel, dentin and cementum) and soft connective tissues (*e.g.*, pulp and periodontal ligaments, including peripheral nerve fibres and blood vessels)^4^,^5^,^6^. The physiological functions of teeth, such as masticatory potential, response to mechanical stress and perceptive potential for noxious stimuli, are efficiently carried out by the characteristic three-dimensional multicellular structure that establishes functional harmonization with the maxillofacial region^2^,^3^. Tooth loss due to dental caries, periodontal disease and traumatic injury causes fundamental oral and general health problems related to oral function and associated general health issues. To restore occlusal function or address aesthetic problems after tooth loss, conventional dental therapies that replace the tooth with artificial materials, such as fixed dental bridges and removable dentures, are commonly used^7^,^8^. Recently, osseo-integrated dental implants, which can restore occlusal function without injuring the adjacent teeth, have been used to treat tooth loss^9^,^10^. Although these artificial therapies have been widely applied in dental rehabilitation, further technological developments based on biological findings are necessary to restore the physiological functions of teeth^6^.

Substantial advances in regenerative technologies have been based on work conducted in many research fields, including developmental biology, stem cell biology and tissue engineering^11^,^12^,^13^,^14^,^15^. Attractive regenerative therapies that can repair local sites of tissue and organ damage have been reported, including stem cell transplantation, cytokine therapy and two-dimensional cell-sheet technology^11^,^16^,^17^,^18^,^19^. Whole-organ replacement therapy has great potential to serve as an ultimate regenerative strategy based on the reconstruction of a fully functional bioengineered organ using three-dimensional cell manipulation *in vitro*^6^,^20^. To regenerate ectodermal organs, including the tooth, hair follicle and salivary gland, a novel concept has been proposed in which a bioengineered organ is generated from bioengineered organ germ by reproducing the developmental process^20^,^21^,^22^,^23^,^24^. A fully functional bioengineered tooth replacement with adequate structure, masticatory function, responsiveness to mechanical stress and perceptive potential for noxious stimulation was recently demonstrated in a murine tooth-loss model^21^,^22^. It is therefore anticipated that whole-tooth replacement therapy will be established in the near future as a novel treatment that will contribute to functional recovery and satisfies aesthetic and physiological requirements^25^.

Donor-organ transplantation is an essential method for replacing a dysfunctional organ and restoring organ function *in vivo*^26^,^27^. Avoidance of immunological rejection following organ transplantation is an important aspect of engraftment and functional recovery of the transplanted organs^28^,^29^. Graft-versus-host disease (GVHD), which may occur during blood transfusion therapy, is one of the clinical complications associated with organ transplantation^30^. Palliative treatments for GVHD currently involve immunosuppressive agents and steroids; however, adequate clinical effects cannot be obtained^31^. Ideally, immunological problems with transplanted tissues or organs would be prevented by autologous transplantation (that is, by using the patient’s own tissue), and utilizing stem cells derived from patients is currently the first choice in regenerative therapies, including stem cell transplantation and tissue engineering^17^,^18^,^19^. In the dental field, autologous tooth or tooth germ transplantation, which has been conventionally performed for many decades, has allowed for successful tooth engraftment in the oral cavity and the restoration of physiological tooth function without immunological rejection^32^,^33^,^34^,^35^. Therefore, it is expected that future whole-tooth restoration in humans will be realized via the autologous transplantation of bioengineered tooth germ reconstructed using a patient’s own stem cells^6^,^22^.

In this study, we demonstrated functional tooth restoration after transplanting bioengineered tooth germ in a postnatal large-animal model. The bioengineered tooth, which was reconstructed using canine permanent tooth germ, developed with the correct tooth structure after autologous transplantation into the jawbone. Furthermore, the erupted bioengineered teeth showed satisfactory physiological function with respect to the biological response to mechanical stress, and this response was equivalent to that of natural teeth. This study highlights the feasibility of fully functional tooth restoration by autologous transplantation of bioengineered tooth germ.

## Results

### Generation of a bioengineered tooth germ

**Figure 1:**
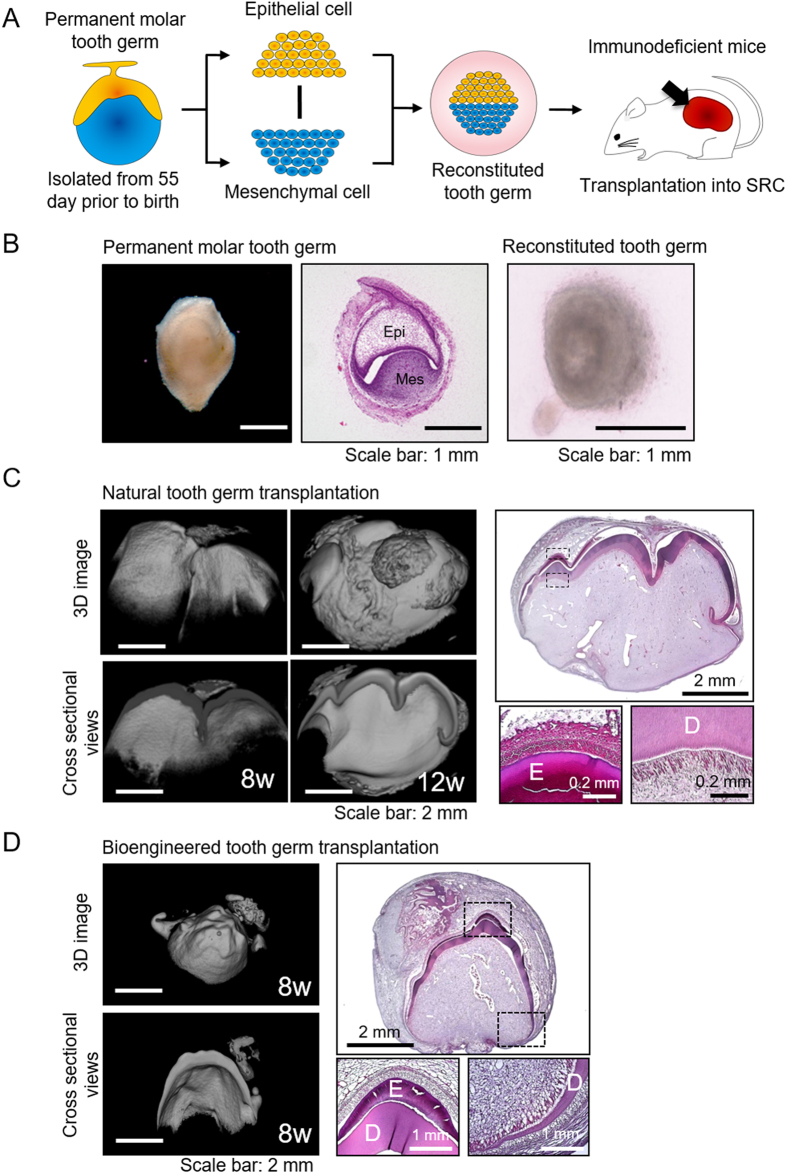
Generation of a bioengineered tooth using embryonic canine tooth germ-derived cells under subrenal capsule transplantation. (**A**) Schematic representation of the generation of reconstituted tooth germ. (Illustration by R.N.) (**B**) Photograph (*left*) and histological image obtained by HE staining (*centre*) of permanent first molar tooth germ (M1) and phase contrast image of reconstituted tooth germ on organ culture day 2 (*right*). Epi, epithelial tissue; Mes, mesenchymal tissue. (**C**) Micro-CT images of natural tooth germ at 8 and 12 weeks after subrenal capsule transplantation. Three-dimensional (3D) images are shown in the upper column, and cross-sectional views are shown in the lower column (*left*). Histological analysis of the bioengineered tooth 12 weeks after subrenal capsule transplantation (*right*). Boxes indicate the area shown at higher magnification in the lower panels. (**E**), enamel; (**D**), dentin. (**D**) Bioengineered tooth germ was reconstituted using single cells derived from molar tooth germ. Micro-CT images (*left*) and histological image (*right*) of the bioengineered tooth 8 weeks after subrenal capsule transplantation. 3D images are shown in the upper column, and cross-sectional views are show in the lower column. Boxes indicate the area shown at higher magnification in the lower panels. (**E**), enamel; (**D**), dentin.

To realize whole-tooth regeneration, previous studies have developed a novel three-dimensional cell manipulation method for developing a bioengineered tooth germ by using embryonic epithelial and mesenchymal cells in mouse models^20^,^21^,^22^. Therefore, we first investigated whether the canine bioengineered tooth germ could be generated in a large-animal model according to our three-dimensional cell manipulation method. We reconstructed the bioengineered tooth germ by using the embryonic tooth germ cells and/or tissues dissected from the maxillary deciduous third molar (dM3) and permanent first molar (M1) of a beagle dog at 55 days prior to birth and performed subrenal capsule transplantation into immunodeficient mice (Fig. 1A,B and Supplemental Fig. 1A,B). Four or eight weeks after transplantation, the bioengineered tooth germ successfully developed the features of a tooth-crown formation, including enamel, dentin and pulp tissue equivalent to that of natural teeth (Fig. 1C,D and Supplemental Fig. 1C). However, the frequency of bioengineered tooth generation was low (16.7%) in the reconstructing condition of epithelial cells and mesenchymal cells (Table 1). The majority of samples in the reconstructing condition of epithelial cells and mesenchymal cells, which did not allow for the development of the bioengineered tooth into a subrenal capsule, did not show tooth tissue structures such as enamel, dentin, pulp and periodontal ligament (PDL) (Supplemental Fig. 2). By contrast, bioengineered tooth generation occurred with a frequency of 100% in the reconstructing conditions of epithelial tissue and mesenchymal tissue, epithelial cells and mesenchymal tissue, and epithelial tissue and mesenchymal cells that were isolated from deciduous third molar (dM3) and permanent first molar (M1) tooth germs (Table 1 and Supplemental Fig. 1). These results indicate that bioengineered tooth germ can be generated using tooth germ cells and/or tissues from a large-animal model; however, the reconstructing condition of epithelial cells and mesenchymal cells was inefficient compared with the other conditions.

### Preparation and optimization of the autologous tooth germ transplantation model

To achieve whole-tooth restoration in humans, it is desirable to autologously transplant bioengineered tooth germ reconstructed using a patient’s own stem cells to prevent immunological rejection, and it is necessary to first establish an autologous tooth germ transplantation system in a large-animal model. We therefore investigated whether the canine bioengineered tooth germ reconstructed using epithelial and mesenchymal components isolated from individual tooth germs could develop after autologous transplantation into the jawbone. We initially analysed natural tooth development in the canine lower jaw, particularly that of the deciduous molars (dM1, dM2, dM3) and permanent premolars (P2, P3, P4), by CT imaging from postnatal days 30 to 210 (Supplemental Fig. 3). At postnatal day 30, all the deciduous molars erupted into the oral cavity (Supplemental Fig. 4A), and all the permanent premolar germs, which were at a developmental stage suitable for the reconstruction of bioengineered tooth germ, were present on the root side of the deciduous molars based on micro-CT and histological analysis (Supplemental Figs 3 and 4B,C). In the development of the permanent premolar germs (P2, P3, P4), initial hard tissue formation of the cusp tip was observed at 30 days after birth, and crown formation was observed at 90 days after birth. Thereafter, all the premolars were successfully replaced with deciduous molars, and natural tooth development was completed until root formation at approximately 210 days after birth (Supplemental Fig. 3). Based on these results, we adopted the autologous transplantation model in dogs at postnatal day 30 to reconstruct bioengineered tooth germ using the permanent premolar germs that were subsequently transplanted into the autologous lower jaw.

### Development and eruption of a canine bioengineered tooth germ by autologous transplantation

**Figure 2:**
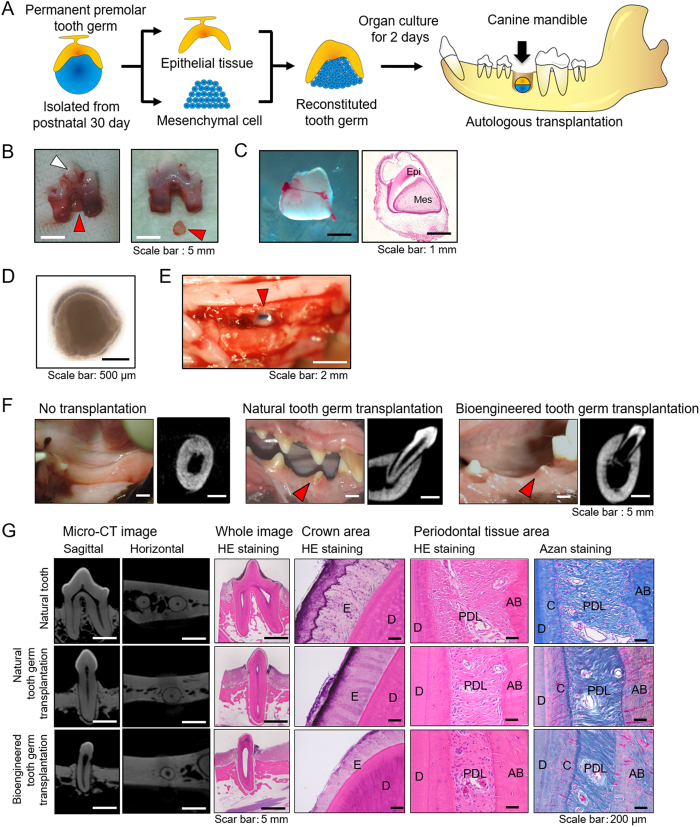
Development of bioengineered tooth using postnatal canine-derived tissue in the autologous transplantation model. (**A**) Schematic representation of autologous transplantation methods for the bioengineered tooth using postnatal canine-derived tissue. (Illustration by R.N.) (**B**) Photograph of the extracted deciduous molar (dM) with permanent premolar tooth germ (*left*) and isolation of the permanent premolar tooth germ (*right*). White arrowhead, extracted deciduous molar; Red arrowhead, permanent premolar tooth germ. (**C**) Photograph (*left*) and histological image by HE staining (*right*) of the isolated permanent premolar tooth germ. Epi, epithelial tissue; Mes, mesenchymal tissue. (**D**) Phase contrast image of the reconstituted canine tooth germ using epithelial tissue and mesenchymal cells derived from permanent premolar tooth germ after 2 days in organ culture. (**E**) Photograph of the autologous transplantation of bioengineered tooth germ into canine lower jawbone. Red arrowhead, bioengineered tooth germ. (**F**) Oral photographs and CT images of the erupted bioengineered tooth at 180 days after transplantation: no transplantation group (*left*), natural tooth germ transplantation group (*centre*), and bioengineered tooth germ transplantation group (*right*). Red arrowhead, erupted tooth. (**G**) Micro-CT images and histological analysis of the natural tooth group (*upper*), the natural tooth germ transplantation group (*middle*), and the bioengineered tooth germ transplantation group (*lower*). Histological analysis of the bioengineered tooth over the crown area and the periodontal tissue area. (**E**) enamel; (**D**) dentin; (**C**) cementum; PDL, periodontal ligament; (**A**,**B**) alveolar bone.

We previously reported that bioengineered tooth germ can successfully develop and erupt into an oral cavity in a murine transplantation model^22^. We next investigated whether canine bioengineered tooth germ could develop and erupt into the oral cavity in a large animal (Fig. 2A). In this canine model, the deciduous molars (dM1, dM2, dM3) were extracted from the lower jaw of a dog at postnatal day 30 (Fig. 2B). The permanent premolar germs (P2, P3, P4) were isolated from the root furcation area of the extracted deciduous molars (Fig. 2B and C), and a bioengineered tooth germ was generated using the reconstructing condition of epithelial tissue and mesenchymal cells (Fig. 2D). Both the natural premolar germs (non-dissected tooth germs) and the bioengineered tooth germs were transplanted into the autologous lower jaw with correct orientation after 2 days in organ culture (Fig. 2E). No tooth eruption occurred in the no-transplantation control, and it was indicated that there were no residual tooth germs or tooth germ tissues in the transplantation area (Fig. 2F). However, a successful tooth eruption into the oral cavity was observed in the case of the natural tooth germ (non-dissected tooth germ) transplantation at 180 days after transplantation (Fig. 2F). Similarly, the crown cusp of the bioengineered tooth was observed by micro-CT analysis in the lower jaw at 60 days after transplantation, and the resulting bioengineered tooth successfully erupted into the oral cavity at 180 days after transplantation (Fig. 2F and Supplemental Fig. 5). The developmental process of the bioengineered tooth after transplantation was practically identical to that of a natural tooth (Supplemental Figs 3 and 5). Micro-CT and histological analyses revealed that the bioengineered tooth had the correct tooth tissue structure and a single root shape composed of enamel, dentin, cementum and periodontal ligament (Fig. 2G). These results indicated that bioengineered tooth reconstruction from canine permanent tooth germs could develop the proper tooth structures after autologous transplantation into the jawbone.

### Structural analysis of a canine bioengineered tooth

**Figure 3:**
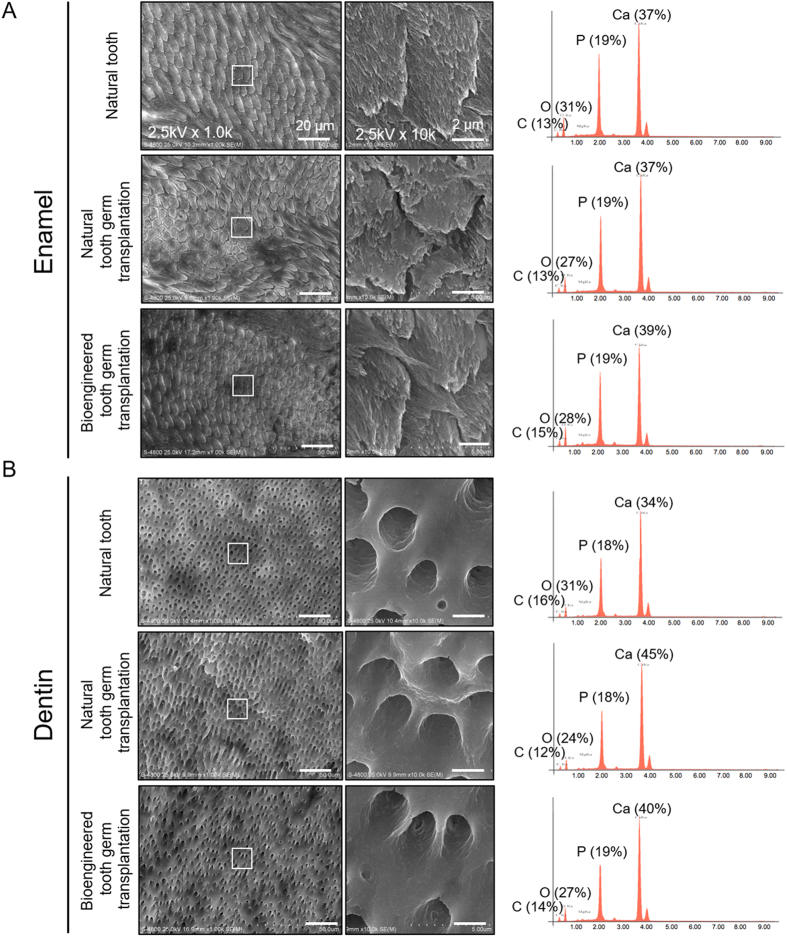
SEM and EDX analysis in the bioengineered tooth. SEM image of enamel (**A**) and dentin (**B**) of the natural tooth, the erupted tooth formed by the transplantation of natural tooth germ and the bioengineered tooth. Boxes indicate the area shown at higher magnification in the centre panels. To analyse the structure of the enamel rod and dentin tube, the tooth was treated with 40% phosphoric acid for 10 sec and sodium hypochlorite for 15 sec. The surface composition of each tooth was analysed by EDX. C, carbon; O, oxygen; P, phosphorus; Ca, calcium.

Tooth hard tissues, including enamel and dentin, have a distinctive ultrastructure that provides both strength and function in the severe environment of the oral cavity^2^,^3^. Thus, we performed scanning electron microscopy (SEM) and energy-dispersive X-ray (EDX) spectroscopy to evaluate the ultrastructure of the bioengineered tooth. The bioengineered tooth had the correct ultrastructure of tooth hard tissue, such as enamel rods and dentinal tubes, as did the natural tooth and the erupted tooth after natural tooth germ transplantation (Fig. 3A,B). Furthermore, EDX analysis revealed that the specific elements found in the enamel and dentin, including carbon (C), oxygen (O), phosphorus (P) and calcium (Ca), were detected at the same frequency in the natural tooth, the erupted tooth after natural tooth germ transplantation and the bioengineered tooth (Fig. 3A,B). These results indicated that the canine bioengineered tooth formed the correct tooth architecture with the same components as the natural tooth after the autologous transplantation of bioengineered tooth germ into the jawbone.

### Functional analysis of the periodontal ligament of the canine bioengineered tooth

**Figure 4:**
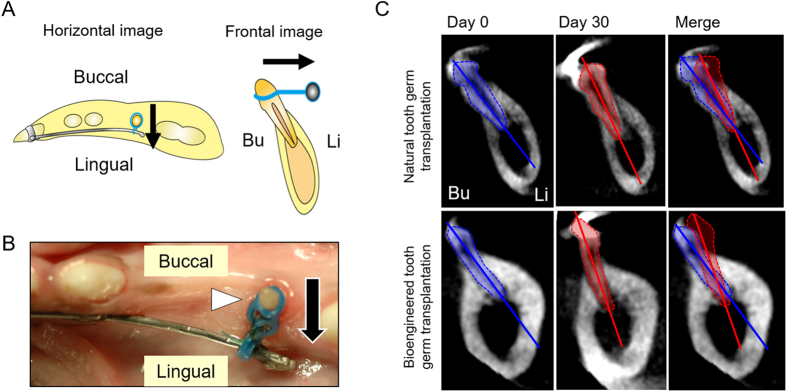
Experimental tooth movement in the canine model. (**A**) Schematic representation of orthodontic movement of the natural tooth, the erupted tooth formed by transplantation of natural tooth germ and the bioengineered tooth. (Illustration by R.N.) (**B**) Oral photograph of the orthodontic appliance designed for the canine jawbone. The erupted teeth were continuously loaded with 10 gf of horizontal orthodontic force (from the buccal side to the lingual side) for 30 days using an orthodontic appliance. Arrowhead, erupted tooth; arrow, direction of orthodontic force. (**C**) CT images of the tooth movement of the erupted tooth formed by transplantation of natural tooth germ and the bioengineered tooth before orthodontic treatment (*left panels*, blue) and after orthodontic treatment (*centre panels*, red). Merged images before and after orthodontic treatment are shown (*right panels*).

The PDL plays an important role in physiological tooth function, such as absorption of occlusal loading, maintenance of alveolar bone height and orthodontic tooth movement accompanied by bone remodelling^2^,^36^. Thus, we investigated whether an erupted bioengineered tooth could respond to mechanical force as a proxy for physiological periodontal function. To analyse the PDL function of the bioengineered tooth, 10 gf of orthodontic force was applied to the bioengineered tooth for 30 days using an orthodontic treatment device (Fig. 4A,B). As a result, the bioengineered tooth moved in response to the orthodontic force in a manner similar to that of the erupted tooth generated by natural tooth germ transplantation (Fig. 4C). These results demonstrated that the PDL of the canine bioengineered tooth successfully mediated tooth movement in response to mechanical stress without ankylosis.

## Discussion

In this study, we demonstrated successful tooth restoration by autologous transplantation of bioengineered tooth germ into a tooth loss region in a postnatal canine model. We also determined that the bioengineered tooth erupted into the oral cavity with the features of proper tooth tissue formation and restored physiological tooth function, such as the response to orthodontic mechanical force. This study represents a substantial advancement in organ replacement therapy through the transplantation of bioengineered organ germ as a practical model for future whole-organ regeneration.

Whole-tooth replacement therapy holds great promise for the replacement of lost teeth by reconstructing a fully functional bioengineered tooth using three-dimensional cell manipulation *in vitro*^6^,^20^. It is anticipated that bioengineering technology will ultimately enable the reconstruction of fully functional organs *in vitro* through the proper arrangement of epithelial and mesenchymal cell components. Many researchers have attempted to generate bioengineered tooth germ using epithelial and mesenchymal cells from embryonic tooth germ^37^,^38^ or postnatal tooth germ^39^,^40^,^41^,^42^,^43^,^44^ from various species, including mice, rats and swine. With the goal of precisely replicating the developmental processes that occur in organogenesis, the study of an *in vitro* three-dimensional cell manipulation method called the bioengineered organ germ method has been recently reported^20^,^21^,^22^,^23^,^24^. However, additional evidence of the practical application to human medicine is required to demonstrate the generation of bioengineered tooth germ using postnatal cell sources in a large-animal model^22^. In this study, we demonstrated the successful generation of bioengineered tooth germ reconstructed using epithelial tissue and mesenchymal cells isolated from deciduous tooth germs or permanent tooth germs in a diphyodont mammalian model. In the case of reconstruction using epithelial cells and mesenchymal cells, a bioengineered tooth germ developed at a low frequency. These results suggested that the reconstructing condition of epithelial cells and mesenchymal cells was inefficient compared with the other conditions. Zhang W. *et al*. reported that the frequency of tooth germ reconstruction was influenced by critical causes regarding the cell seeding density or insufficient direct contact with epithelial and mesenchymal tooth germ cells^44^. Furthermore, certain factors, such as distinctive gradual tissue development (*i.e.,* developmental speed) in large animals, might reduce the generation rate of bioengineered tooth germ during the tissue organization process reconstructed from single cells^45^. This advancement is significant for the concept of whole-tooth replacement therapy, in which a bioengineered tooth germ can be reconstructed utilizing the bioengineered organ germ method and postnatal stem cells.

To repair local sites of tissue and organ damage, a current regenerative concept involves stem cell transplantation or cell-sheet engineering using purified tissue-derived stem cells or pluripotent stem cells^11^,^16^,^46^. In the dental field, basic research on stem/progenitor cells has provided new insights concerning tooth tissue-derived stem cells, and these cells contribute to stem cell-mediated tissue repair, including dentin, pulp and periodontal tissue regeneration^47^,^48^. Stem cell transplantation therapy has essentially focused on the use of a patient’s own stem cell source because preventing immunological rejection is a critical issue for graft survival and the recipient’s safety^49^,^50^,^51^. In dentistry, autologous transplantation of a tooth or tooth germ is now available for biological dental treatment against tooth loss^33^,^34^,^35^. These treatments could allow for successful engraftment into the oral cavity and restore physiological tooth function, and the use of an autologous tooth/tooth germ could prevent immunological rejection after transplantation compared with allogeneic tooth transplantation^33^,^34^,^35^. Therefore, from the perspective of medical safety, it is desirable to perform autologous transplantation of a bioengineered organ reconstructed using a patient’s own stem cells/organs to prevent an immunological response^49^,^50^,^51^. However, autologous transplantation of a natural tooth/tooth germ is limited by the number of available teeth and the size of a given type of tooth. In our present study, we demonstrated successful tooth restoration after autologous transplantation of bioengineered tooth germ reconstructed using autologous epithelial and mesenchymal tooth germ cells in a large-animal model. It was also reported that a bioengineered tooth could optimize the tooth size by regulating the contact length of epithelial cells and mesenchymal cells^52^. This study demonstrates the feasibility of practical tooth replacement therapy by the transplantation of bioengineered tooth germ as an alternative treatment for autologous tooth transplantation.

For the realization of whole-tooth replacement therapy, a regenerated tooth developed from bioengineered germ must be capable of acquiring full functionality, including masticatory performance and biological responses to mechanical stress in the maxillofacial region^21^,^22^,^25^. The tooth is a characteristic calcified tissue structure with adequate hardness and efficient microstructures (*e.g.,* enamel rod and dentinal tubule) that contributes to occlusal stability, food mastication and aesthetics^2^,^3^. Periodontal tissue is composed of the cementum, PDL and alveolar bone, and it establishes a biological connection by inserting the PDL fibre into the cementum and the alveolar bone during root formation^2^,^3^. The structural properties of periodontal tissue play important roles in physiological tooth function, including the absorption of occlusal loading, the maintenance of alveolar bone height and orthodontic tooth movement accompanied by bone remodelling^2^,^36^. We previously reported that a fully functional bioengineered tooth could be developed by transplanting a bioengineered organ germ, which restored physiological tooth function in the maxillofacial region^21^,^22^. In this study, we demonstrated that a bioengineered tooth reconstructed from canine permanent tooth germ reproduced the correct tooth structure, including calcified components and enamel and dentin microstructure. Furthermore, the erupted bioengineered tooth had a single-root shape with the proper periodontal tissue structure, and it achieved physiological tooth function in terms of biological response to mechanical stress equivalent to the PDL function of a natural tooth. This study shows that transplantation of bioengineered tooth germ has potential as a biological dental treatment that can result in essential functional recovery of lost teeth to satisfy aesthetic and physiological requirements.

To address the future clinical application of bioengineered tooth replacement therapy, it is important to identify appropriate cell sources. At present, an immature wisdom tooth (third molar) germ in a young patient is considered a potential candidate for reconstruction of bioengineered tooth germ. It is well known that human wisdom tooth germ begins to mineralize at 7 to 10 years old; therefore, epithelial/mesenchymal stem cells, which can reproduce tooth germ development, are available in the postnatal jawbone^2^. In clinical cases of congenital or accidental tooth/tooth germ loss during jawbone growth, these stem cells derived from wisdom tooth germ have great potential for use in young patients. This study demonstrated whole-tooth replacement by using postnatal tooth germ cells, assuming tooth loss for young patients. If a large-scale culture of epithelium/mesenchymal tooth germ cells were to be established in future, this bioengineered tooth technology would be able to treat a large number of missing teeth. Elderly patients, however, do not have a developing tooth germ that can be used for the reconstruction of bioengineered tooth germ in the patient’s own jaw. In the dental field, recent stem cell biology studies have led to the identification of dental stem cells based on tooth organogenesis for tooth tissue regeneration and tooth regenerative therapy^25^,^47^. Although these stem cells would be valuable cell sources for stem cell transplantation therapy aimed toward dental tissue regeneration, the tooth inductive potential cells, which can replicate an epithelial-mesenchymal interaction for whole-tooth replacement, has not yet been identified^6^. Pluripotent stem cells, including ES cells and iPS cells, are also candidate cell sources that are capable of differentiating into endodermal, ectodermal and mesodermal cells^46^. Recently, sources of iPS cells have been established, including several oral tissues such as pulp, PDL, gingiva and oral mucosa^46^,^53^; these cells can differentiate into dental epithelial and mesenchymal cells^54^,^55^. Further studies that can identify tooth-inducible stem cells in elderly patients for the reconstitution of a bioengineered tooth germ are necessary to realize whole-tooth regenerative therapy in the clinic.

In conclusion, our study demonstrated functional whole-tooth restoration by autologous transplantation of bioengineered tooth germ in a postnatal large-animal model. This study represents a significant advancement in organ replacement therapy through the transplantation of bioengineered organ germ as a practical model for future clinical regenerative medicine.

## Methods

### Study design

This study was designed to demonstrate whether a fully functional tooth bioengineered using postnatal stem cells can be developed in a large-scale animal. Tooth germs of mandibular premolar were dissected from 30-day-old beagle dogs to generate the bioengineered tooth germ using our previously reported organ-germ culture method. First, to evaluate whether the bioengineered tooth germ could develop normally, subrenal capsule transplantation was performed in immunodeficient mice after two days of organ culture; the mice were analysed histologically 4, 8 and 12 weeks after transplantation. Next, canine bioengineered tooth germs were reconstructed using epithelial tissue and mesenchymal single cells derived from permanent premolar tooth germs of 30-day postnatal dogs; the germs were autologously transplanted into the alveolar bone socket in the mandible after two days of organ culture. The bioengineered tooth was analysed radiologically by micro-CT, histologically by haematoxylin-eosin (HE) and Azan staining and morphologically by scanning electron microscopy. Finally, an experimental tooth movement model was used to evaluate the proper periodontal ligament function of the bioengineered tooth.

### Ethics statement of animal research

The study was performed on 6-week-old female immunodeficient mice (Balb/c nu/nu; CLEA, Tokyo, Japan) and beagle dogs at 55 days prior to birth and at postnatal day 30 (Toyo-beagle; ORIENTAL YEAST Co., Ltd., Tokyo, Japan). All animals were handled according to protocols and guidelines approved by the animal committee of Okayama University (OKU-2012334, OKU-2012419) and according to the principles of the Declaration of Helsinki. Mice were operated on under general anaesthesia induced by intraperitoneal injection of 0.4 mL/kg of 1:1 ketamine hydrochloride (Ketalar 500 mg; Daiichi Sankyo Propharma Co., Ltd., Tokyo, Japan) and xylazine (Selactar 2% injection; Bayer HealthCare, Tokyo, Japan). Canines were anesthetized via an intramuscular injection of a mixture of xylazine (8 mg/kg; Bayer HealthCare) and ketamine (80 mg/kg; Daiichi Sankyo Propharma Co., Ltd.). Local anaesthesia with 2% xylocaine containing 1/80,000 epinephrine was additionally provided before bioengineered tooth germ transplantation. The canines were kept in single cages with water and nonsolid food.

### Reconstitution of bioengineered tooth germ

In a previous study, we developed a novel three-dimensional cell manipulation method for forming a bioengineered tooth germ—designated the “organ germ method”—in a mouse model^20^. To clarify whether the canine bioengineered tooth germ could be generated in a large-animal model according to the organ germ method, we first performed verification experiments by using embryonic tooth germ cells and/or tissues dissected from the maxillary deciduous third molar (dM3) and permanent first molar (M1) of a beagle dog at 55 days prior to birth (Fig. 1, Supplemental Figs 1 and 2). When we autologously transplanted the bioengineered tooth germs into the oral cavity, tooth germs of the mandibular permanent second (P2), third (P3) and fourth (P4) premolars were dissected from beagle dogs at postnatal day 30 (Fig. 2 and Supplemental Fig. 4). The epithelial and mesenchymal tissues were separated from the dissected germ by treatment with 1.2 U/mL Dispase II (BD, Franklin Lakes, NJ, USA) and 20 U/mL deoxyribonuclease I (DNase I; Takara Bio, Shiga, Japan) for 12.5 min. To obtain single mesenchymal cells, mesenchymal tissues were treated once with 0.25% trypsin (Sigma, St. Louis, MO, USA), 50 U/mL collagenase I and 20 U/mL DNase I for 10 min at 37 °C; twice with 100 U/mL collagenase I (Worthington, Lakewood, NJ, USA) for 10 min at 37 °C; and once with 0.25% trypsin and 20 U/mL DNase I for 5 min at 37 °C. Similarly, epithelial tissues were treated with 50 U/mL collagenase I for 20 min at 37 °C and then with 0.25% trypsin and 20 U/mL DNase I for 5 min at 37 °C to obtain single epithelial cells. Bioengineered tooth germs were reconstituted using our previously described three-dimensional cell manipulation technique (organ germ method)^20^. The intact epithelial/mesenchymal tissues and the epithelial/mesenchymal single cells (2.0 × 10^7^ cells/mL each in one of the bioengineered tooth germ) were prepared to evaluate the generation rate of tooth germ reconstruction. The bioengineered tooth germs were reconstructed in four combinations; (1) epithelial tissue & mesenchymal tissue, (2) epithelial cells & mesenchymal tissue, (3) epithelial tissue & mesenchymal cells and (4) epithelial cells & mesenchymal cells (Table 1 and Supplementary Fig. 1). In the reconstruction of epithelial and mesenchymal cells according to the organ germ method, we used 2.0 × 10^7^ cells/mL epithelial and mesenchymal cells each to generate a bioengineered tooth germ (Fig. 1B). However, in the reconstruction of each tissue and cells combination, epithelial or mesenchymal tissue was placed in type-I collagen gel (Cellmatrix Type I-A, Nitta Gelatin Inc., Osaka, Japan). Thereafter, an epithelial or mesenchymal cell pellet (2.0 × 10^7^ cells/mL) was placed in the same collagen gel and made contact with the existing epithelial or mesenchymal tissue (Supplementary Fig. 1A). These bioengineered tooth germs were cultured on a cell culture insert (0.4 μm pore diameter; BD) in basal medium consisting of α-MEM (Life Technologies, Gaithersburg, MD, USA), 10% foetal bovine serum (FBS; Life Technologies), 100 μM of L-ascorbic acid 2-phosphate (WAKO, Tokyo, Japan), 100 U/mL of penicillin and 100 μg/mL of streptomycin (SIGMA, St. Louis, MO, USA) at 37 °C in 5% CO_2_ for 2 days.

### Transplantation of bioengineered tooth germ

To evaluate whether the reconstituted tooth germ could develop, subrenal capsule transplantation of the natural tooth germ was performed, and tooth germs were reconstituted in several combinations into 6-week-old female immunodeficient mice (CLEA) after 2 days of organ culture. Four weeks after transplantation in the reconstruction of each tissue and cell combination (*i.e.*, epithelial tissue and mesenchymal tissue, epithelial cells and mesenchymal tissue, epithelial tissue and mesenchymal cells) and 8 or 12 weeks after transplantation in the reconstruction of each cell and cell combination (*i.e*., epithelial cells & mesenchymal cells), the bioengineered teeth were harvested from the immunodeficient mice and then analysed histologically.

Next, we developed a method for autologous transplantation of natural (non-dissected) tooth germ and bioengineered tooth germ in a postnatal canine model. Canine permanent premolar (P2, P3 and P4) tooth germs were dissected from the mandible of beagle dogs at postnatal day 30, and bioengineered tooth germ consisting of epithelial tissue and mesenchymal cells was then generated. After 2 days of organ culture of the natural and bioengineered tooth germs, the germs were autologously transplanted into the alveolar bone socket of the same mandible from which the tooth germs were isolated (Fig. 2A–E). At 6 months (180 days) after transplantation, the erupted natural tooth and bioengineered tooth were harvested from the canine mandible and analysed radiologically, histologically and morphologically. To evaluate the function of the periodontal ligament (PDL), several samples of the transplanted natural tooth and the bioengineered tooth were submitted to orthodontic experiments at 180 days after natural or bioengineered tooth germ transplantation, when these teeth had erupted into the oral cavity.

### Computed tomography analysis

To analyse natural and bioengineered tooth development and experimental tooth movement in the canine mandible, computed tomography (CT) images were obtained using a PLANMECA ProMax 3D Max (PLANMECA, Helsinki, Finland) and the accompanying analysis software. Micro-CT images of the collected bioengineered teeth were obtained using a SkyScan 1174 compact micro-CT (BRUKER, Aartselaar, Belgium). CT scans were captured at a resolution of 64 μm, in which 269 sections were reconstructed to produce the final images using SkyScan software.

### Histological analysis

Collected samples were fixed in 4% paraformaldehyde (PFA) for 3 days and decalcified with formic citric acid for 60 days. The paraffin sections were stained with standard haematoxylin and eosin (HE), Azan and toluidine blue. For the histological analysis of enamel, fixed tissues were embedded in methyl-methacrylate (MMA) resin, and undecalcified 30-μm-thick sections were obtained with a micro-cutting machine. MMA resin sections were analysed histologically after HE or Azan staining.

### Scanning electron microscopy and energy-dispersive X-ray spectroscopy

The bioengineered teeth were fixed with 1% formaldehyde and 1% osmium tetroxide for 15 min each. Fixed samples were cut longitudinally and treated with 40% phosphoric acid for 10 sec and sodium hypochlorite for 15 sec. Finally, samples were sputter-coated with osmium plasma, and images were obtained using a scanning electron microscope (SEM: S-4800 Type2, HITACHI Ltd., Tokyo, Japan). The composition of the bioengineered tooth surface was analysed using the energy-dispersive X-ray spectroscopy instrument (EMAX ENERGY EX-350, HORIBA Ltd., Kyoto, Japan) attached to the SEM (S-4800 Type2).

### Experimental orthodontic treatments

To evaluate the PDL function of the bioengineered teeth, several samples of the transplanted natural teeth and the bioengineered teeth were submitted to orthodontic experiments 180 days after natural or bioengineered tooth germ transplantation, when these teeth had erupted into the oral cavity. The erupted teeth were continuously loaded with 10 gf of horizontal orthodontic force (from the buccal side to the lingual side) for 30 days using an orthodontic appliance (Fig. 4A,B). The orthodontic force was measured by using a tension gauge (Mitutoyo Corporation, Kanagawa, Japan). CT scans were performed to analyse tooth movement using the ProMax 3D CT-machine both before (day 0) and after (day 30) orthodontic treatment.

### Statistical analysis

Statistical analyses were performed using the chi-square test, and p-values less than 0.05 were considered to be statistically significant. Analyses were performed using JMP (version 10.0; SAS Institute Inc., NC, USA). *P < 0.001 (chi-square test).

## Additional Information

**How to cite this article:** Ono, M. *et al*. Practical whole-tooth restoration utilizing autologous bioengineered tooth germ transplantation in a postnatal canine model. *Sci. Rep.* **7**, 44522; doi: 10.1038/srep44522 (2017).

**Publisher's note:** Springer Nature remains neutral with regard to jurisdictional claims in published maps and institutional affiliations.

## Supplementary Material

Supplemental Information

## Figures and Tables

**Table 1:** Frequency of bioengineered tooth germ development under the various reconstructing conditions.

Reconstructing condition	Frequency of reconstructed tooth germ development
Epithelial tissue and Mesenchymal tissue	100% (6/6)
Epithelial tissue and Mesenchymal cells	100% (6/6)
Epithelial cells and Mesenchymal tissue	100% (6/6)
Epithelial cells and Mesenchymal cells	16.7% (3/18)*

*P < 0.001 (chi-square test).
