# Supplementary material for: Practical whole-tooth restoration utilizing autologous bioengineered tooth germ transplantation in a postnatal canine model
Source: Sci Rep. 2017 Mar 16;7:44522. doi: 10.1038/srep44522 (PMC5353657; doi:10.1038/srep44522)
Supplement: Supplemental Information [file srep44522-s1.pdf]

SUPPLEMENTARY INFORMATION

Practical whole-tooth restoration utilizing autologous bioengineered tooth germ transplantation in a postnatal canine model

Mitsuaki ONO, PhD[1,2*,] Masamitsu OSHIMA, PhD[1,3,4*], Miho OGAWA, PhD[3,4,5], Wataru SONOYAMA, PhD[1], Emilio Satoshi HARA, PhD[1], Yasutaka OIDA, PhD[1], Shigehiko SHINKAWA, PhD[1], Ryu NAKAJIMA, PhD[1], Atsushi MINE, PhD[1], Satoru HAYANO, PhD[6], Satoshi FUKUMOTO, PhD[7], Shohei KASUGAI, PhD[8], Akira YAMAGUCHI, PhD[9,10], Takashi TSUJI, PhD[3,4,5] & Takuo KUBOKI, PhD[1†]

[1]Department of Oral Rehabilitation and Regenerative Medicine, Graduate School of Medicine, Dentistry and Pharmaceutical Sciences, Okayama University, Okayama, 700-8525, JAPAN
[2]Department of Molecular Biology and Biochemistry, Graduate School of Medicine, Dentistry and Pharmaceutical Sciences, Okayama University, Okayama, 700-8525, JAPAN
[3]Research Institute for Science and Technology, Tokyo University of Science, Noda, Chiba, 278-8510, JAPAN
[4]RIKEN Center for Developmental Biology, Kobe, Hyogo, 650-0047, JAPAN
[5]Organ Technologies Inc., Tokyo, 105-0001, JAPAN
[6]Department of Orthodontics, Graduate School of Medicine, Dentistry and Pharmaceutical Sciences, Okayama University, Okayama, 700-8525, JAPAN
[7]Division of Pediatric Dentistry, Tohoku University Graduate School of Dentistry, Sendai, Miyagi, 980-8575, JAPAN
[8]Section of Oral Implantology and Regenerative Dental Medicine, Graduate School of Tokyo Medical and Dental University, Bunkyo-ku, Tokyo, 113-8549, JAPAN
[9]Section of Oral Pathology, Department of Oral Restitution, Graduate School of Tokyo Medical and Dental University, Bunkyo-ku, Tokyo, 113-8549, JAPAN
[10]Oral Health Science Center, Tokyo Dental College, Chiyoda-ku, Tokyo, 101-0061, JAPAN

[*]These authors contributed equally to this work.
[†]To whom correspondence may be addressed: Takuo Kuboki, DDS, PhD.

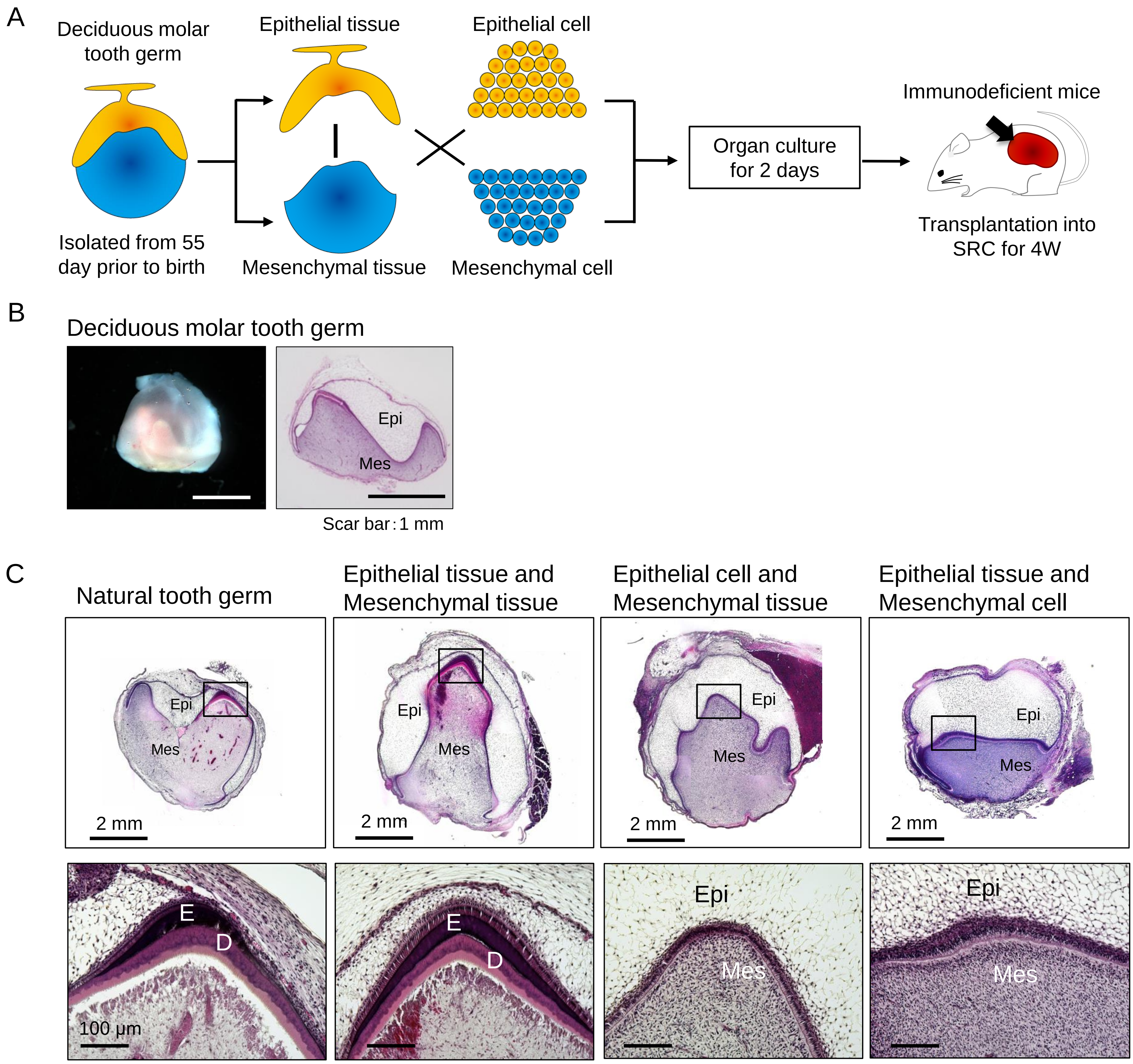

Supplemental Figure 1, Ono *et al.*

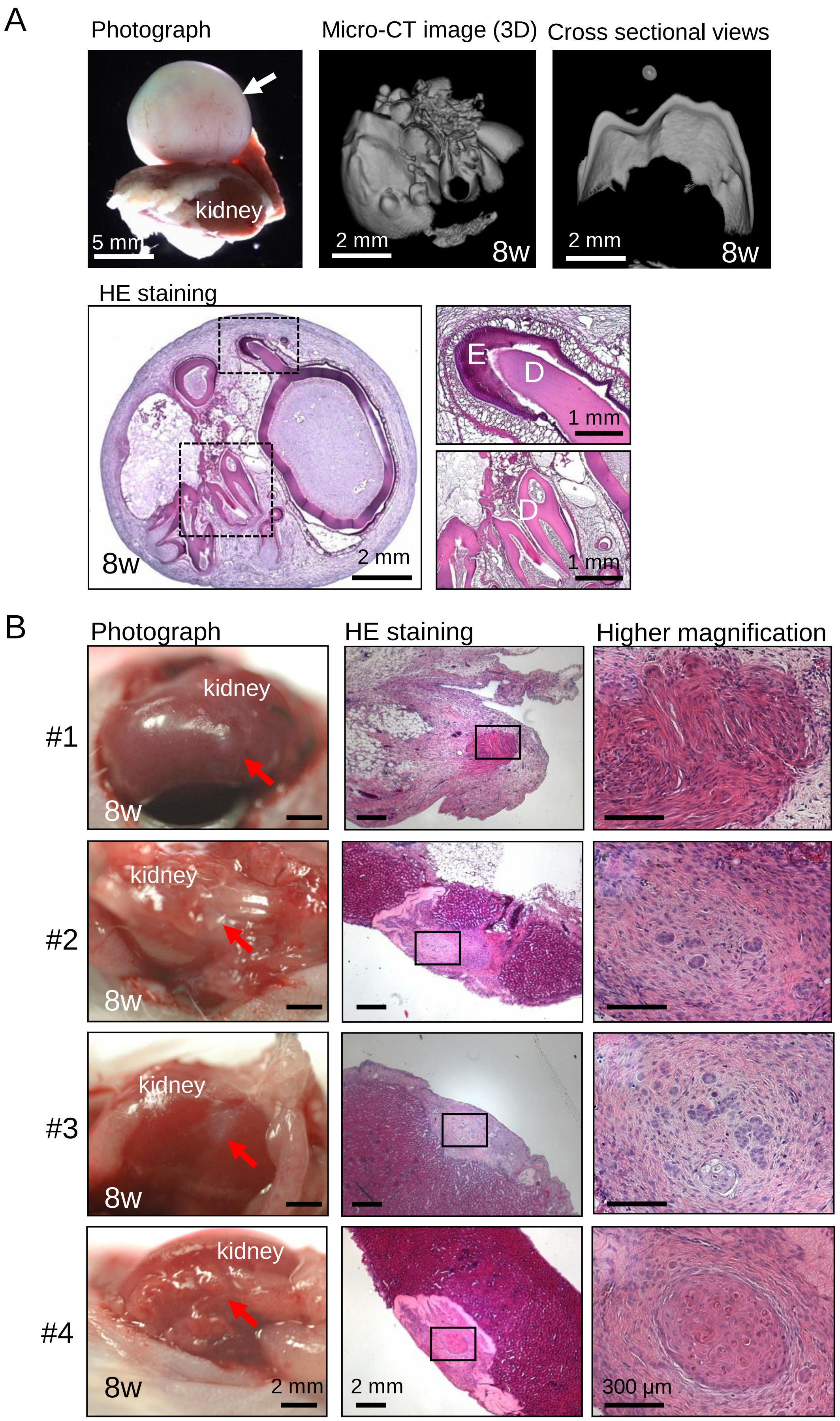

Supplemental Figure 2, Ono *et al.*

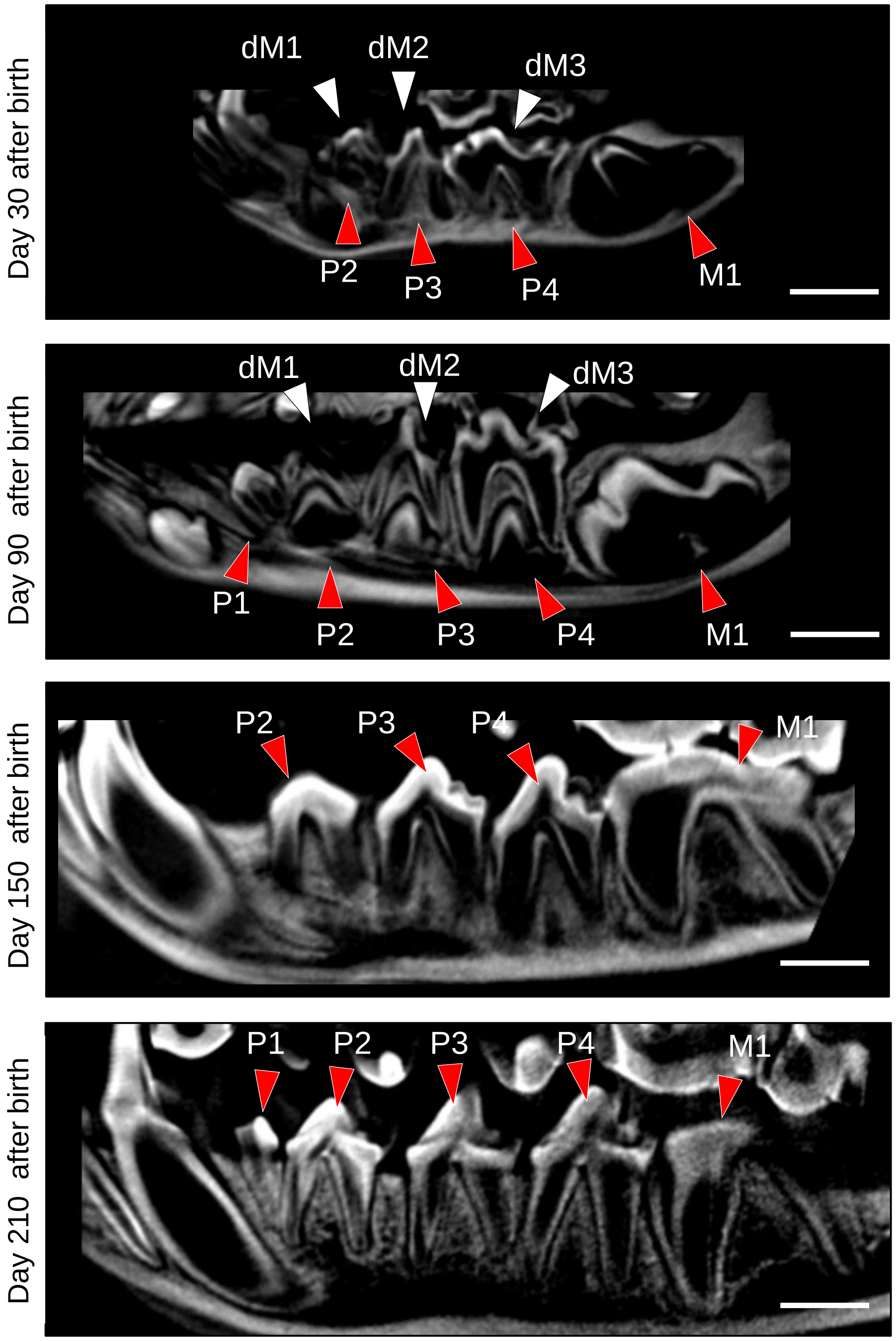

Scale bar: 10 mm

Supplemental Figure 3, Ono *et al.*

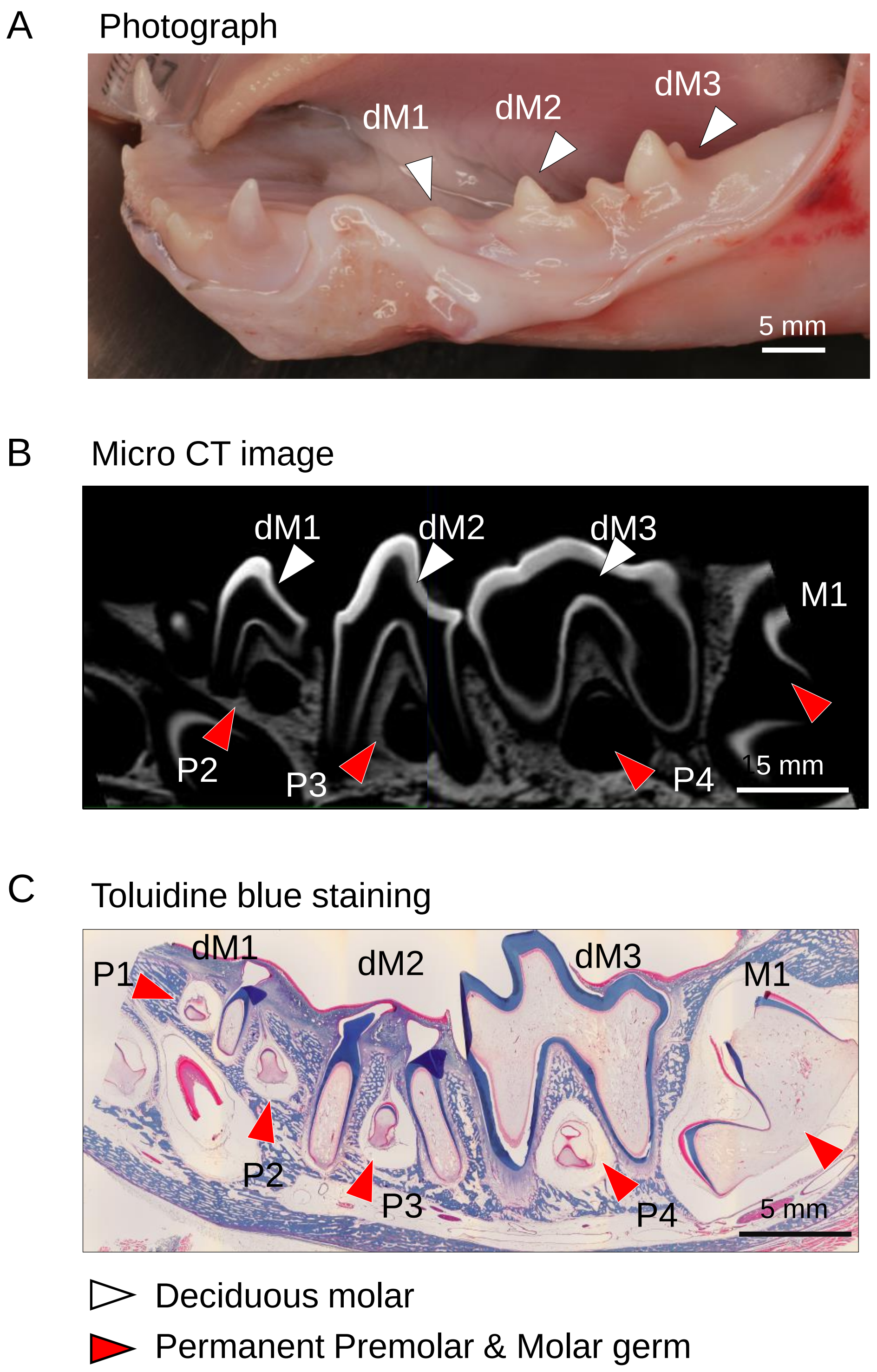

Supplemental Figure 4, Ono *et al.*

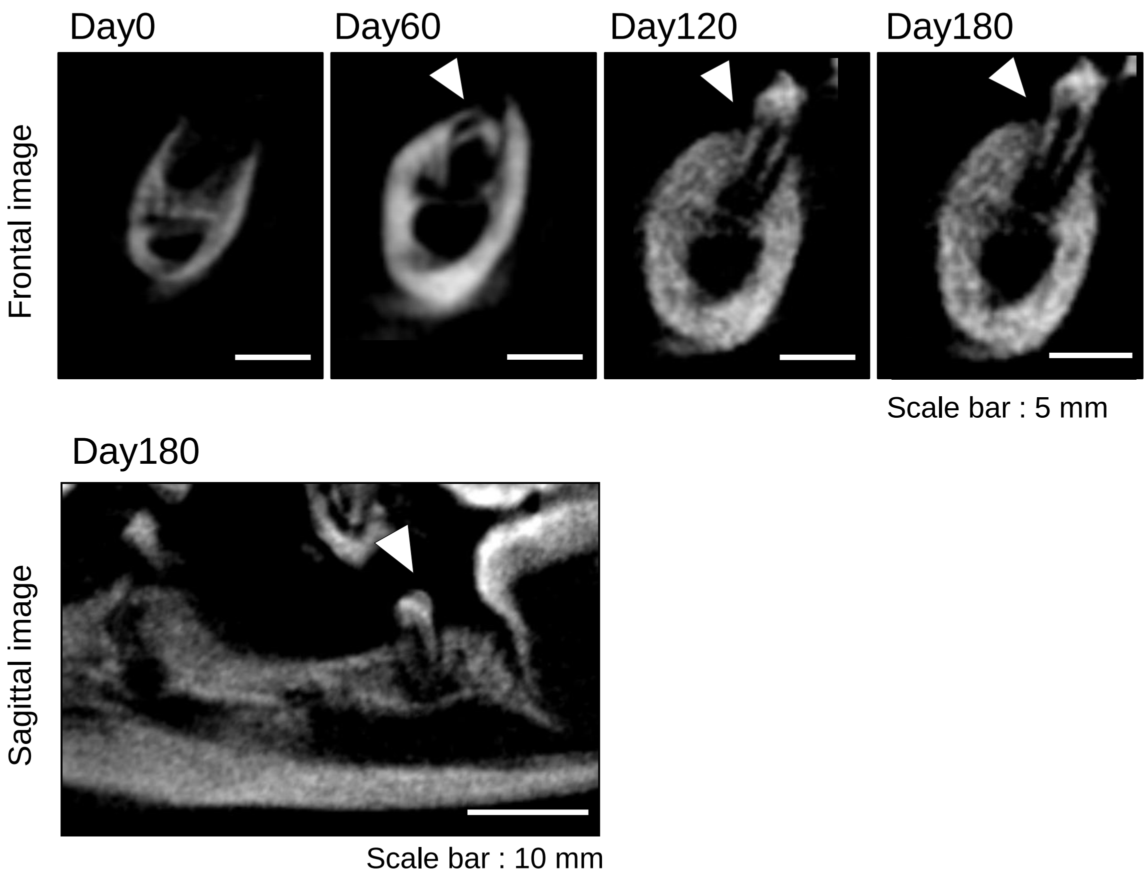

Supplemental Figure 5, Ono *et al.*

Legends to Supplemental Figures

Supplemental Fig. 1. Generation of the bioengineered tooth under the various reconstructing conditions.
(A) Schematic representation of various reconstructing conditions for bioengineered tooth germ. (Illustration by R.N.) (B) Photograph and histological image of deciduous molar tooth germ. Epi, epithelial tissue; Mes, Mesenchymal tissue. (C) Histological analysis of the bioengineered tooth at 4 weeks after subrenal capsule transplantation under the various reconstructing conditions. Boxes indicate the area shown at higher magnification in the lower panels. E, enamel; D, dentin; Epi, epithelial tissue; Mes, mesenchymal tissue.

Supplemental Fig. 2. Generation of bioengineered tooth in the reconstructing condition of epithelial cells and mesenchymal cells.
(A) The frequency of bioengineered tooth generation was low (16.7%) in the reconstructing condition of epithelial cells and mesenchymal cells. In a few successful samples, the bioengineered tooth germ developed into subrenal capsule (*upper, white arrow*) and showed tooth-crown formation in micro-CT analysis (*upper*). Histological analysis of the bioengineered tooth showed the tooth tissue structure, including the enamel and dentin, which were equivalent to those of the natural tooth (*lower*). Boxes indicate the area shown at higher magnification. E, enamel; D, dentin.
(B) The majority of samples (83.3%) in the reconstructing condition of epithelial cells and mesenchymal cells did not show the development of the bioengineered tooth into the subrenal capsule (*left panels, red arrow*). These samples did not show tooth tissue structures such as enamel, dentin, pulp or PDL (*centre and right panels*). Boxes indicate the area shown at higher magnification.

Supplemental Fig. 3. CT images at various tooth developmental stages in the canine mandible.
CT images of canine tooth development in the mandible at postnatal days 30, 90, 150 and 210. White arrowhead, deciduous molars (dM1, dM2 and dM3); Red arrowhead, permanent premolars (P1, P2, P3 and P4) and permanent first molar (M1).

Supplemental Fig. 4. Micro-CT and histological analysis of the canine mandible at postnatal day 30.
Photograph (A), micro-CT image (B) and histological image obtained by toluidine blue staining (C) of the canine mandible at postnatal day 30. All deciduous molars (dM1, dM2 and dM3) erupted into the oral cavity, and the permanent premolar germs (P2, P3, and P4) were at a developmental stage suitable for the reconstruction of bioengineered tooth germ.

Supplemental Fig. 5. CT images of the bioengineered tooth development.
Continual CT images of bioengineered tooth development at 0, 60, 120 and 180 days after autologous transplantation into the mandible. White arrowhead, bioengineered tooth.
